# All-inorganic inverse perovskite solar cells using zinc oxide nanocolloids on spin coated perovskite layer

**DOI:** 10.1186/s40580-017-0113-2

**Published:** 2017-07-28

**Authors:** Naoyuki Shibayama, Hiroyuki Kanda, Shin-ichi Yusa, Shota Fukumoto, Ajay K. Baranwal, Hiroshi Segawa, Tsutomu Miyasaka, Seigo Ito

**Affiliations:** 1 Department of Materials and Synchrotron Radiation Engineering, Graduate School of Engineering, University of Hyogo, 2167 Shosha, Himeji, Hyogo 671-2280 Japan; 2 Department of Applied Chemistry, Graduate School of Engineering, University of Hyogo, 2167 Shosha, Himeji, Hyogo 671-2280 Japan; 3 Research Center for Advanced Science and Technology (RCAST), The University of Tokyo, 4-6-1 Komaba, Meguro-ku, Tokyo, 153-8904 Japan; 4 Graduate School of Engineering, Toin University of Yokohama, Yokohama, Kanagawa 225-8503 Japan

**Keywords:** Perovskite solar cell, ZnO nanoparticle, NiO_x_, Spray pyrolysis deposition

## Abstract

We confirmed the influence of ZnO nanoparticle size and residual water on performance of all inorganic perovskite solar cells. By decreasing the size of the ZnO nanoparticles, the short-circuit current density (*Jsc*) and open circuit photovoltage (*Voc*) values are increased and the conversion efficiency is improved. Although the *Voc* value is not affected by the influence of residual water in the solution for preparing the ZnO layer, the *Jsc* value drops greatly. As a result, it was found that it is important to use the oxide nanoparticles with a small particle diameter and to reduce the water content in the oxide forming material in order to manufacture a highly efficient all inorganic perovskite solar cells.

## Background

The rapid development of organic–inorganic metal halide perovskite (CH_3_NH_3_PbI_x_Br_3−x_) based solar cells has attracted worldwide attention due to the material’s high absorption coefficient, small exciton binding energy, and long carrier diffusion length [1–4]. This rapidly increasing conversion efficiency achieving from initial 3.81 to 22.1%, have been realized in period of the 7 years [5–9]. Since the perovskite is an inexpensive material and fabricated solar cell has a simple device structure owing to its solution process, it has the possibility to replace the commercially established solar cell such as Si, CIGS, and CdTe solar cells [10, 11]. In the commonly reported perovskite solar cells, organic materials (Spiro-OMe-TAD, PCBM, C_60_) on top of perovskite active layer having an energy level conforming to the perovskite layer and having good conductivity are used to support the efficient charge collection on top of perovskite active layer [12, 13]. Since these organic materials require high grade purity, they are very expensive. Moreover, they are likely to be the cause of lowering the durability of perovskite solar cells. Accordingly, the development of inexpensive and highly durable materials are required. Researches have been conducted to utilize inorganic materials (CuI, CuSCN, Cu_2_O/CuO) as a novel hole transporting layer (HTL) on the perovskite active layer in place of commonly employed organic layers [14–20].

In recent years, all inorganic based perovskite solar cells using oxide nanoparticles (ZnO, SnO_2_, Zn_2_SnO_4_) as the electron transporting layer (ETL) by using the NiO_x_ layer which in the HTM layer as a scaffold have been reported [21–23]. Although the reported conversion efficiencies were inferior to that of perovskite solar cells using organic materials, all inorganic perovskite solar cells using oxide nanoparticles have been found to exhibit high durability. However, guidelines for improving the performance of all inorganic perovskite solar cells using oxide nanoparticles on the perovskite active layer are still unknown.

In this study, we fabricated all inorganic perovskite solar cells and investigated the influence of oxide nanoparticle size and its role towards solar cell performance. In addition, we investigated the effect of synthesis route on the performance and durability of the fabricated cells. The synthesized ZnO nanoparticles were used as ETL in fabricated all inorganic inverted perovskite solar cell where NiO_x_ layer formed the scaffold. The results of this study can provide guidelines for using nanoparticles in all inorganic perovskite solar cells.

## Experimental

### Materials

All solvents and chemicals were purchased and utilized as obtained. Nickel (II) acetylacetonate, zinc acetate and bathocuproine (BCP) were purchased from Sigma-Aldrich Co. LLC. CH_3_NH_3_PbI_3_-DMF (MAPbI_3_-DMF) was purchased from TCI Co., Ltd. All solvents and reagents were of the highest quality available and were used as received.

### Synthesis of ZnO nanoparticles solution and powders

#### Aqueous process

ZnO nanoparticle_aque was synthesized following protocol in the previously published literature [24]. To synthesize the ZnO nanoparticles by the aqueous process, the 250 μL deionized water was added to 4.46 mmol zinc acetate powder to make solutions. Further 42 μL CH_3_OH was added dropwise for 30 min at room temperature and Zn precursor solution was obtained. In the next step, KOH (7.22 mmol) was dissolved in CH_3_OH (23 mL) to make KOH solution. The Zn precursor solution was refluxed and, KOH solution was added dropwise into the flask maintaining time duration 15 min. Then, the mixture was refluxed for 30 min. The colloidal solution was replaced by isopropanol from water using a rotary evaporator and the concentration of ZnO was adjusted to 0.2 wt% [25–28].

#### Non-aqueous process

Zinc acetate powder (0.2 mmol) was dissolved in benzyl alcohol (50 mL) and it was stirred and maintaining 120 °C until a clear solution was obtained. The mixture was refluxed at three different time intervals of 20 min, 6 and 24 h, at 170 °C, to synthesize the different nanoparticle size of ZnO. The colloidal solution solvent (benzyl alcohol) was replaced with isopropanol using a rotary evaporator and the concentration of ZnO was adjusted to 0.2 wt% [25–28]. The different time interval synthesized ZnO nanoparticle was numbered as, No. 1 of 20 min, No. 2 of 6 h and No. 3 of 24 h, reflux time respectively.

#### Device fabrication

**Fig. 1:**
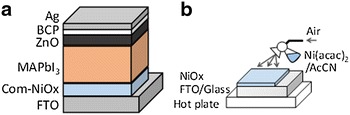
**a** Schematic of all inorganic inverted perovskite solar cell structure of FTO/NiO_x_/MAPbI_3_/ZnO/BCP/Ag. **b** Schematic of NiO_x_ layer preparation method using SPD

An inverse type device comprising FTO/NiO_x_/MAPbI_3_/ZnO/BCP/Ag was fabricated in the steps shown in Fig. 1a. The FTO glass substrates (NSG-Pilkington) were cleaned ultrasonically with detergent, distilled water, and ethanol respectively, and treated with O_3_ for 15 min to eliminate organic impurities and make the surface clean. The substrate arrangements were coated with a dense NiO_x_ compact layer by spray pyrolysis deposition (SPD) method using a diluted solution of Nickel acetate acetylacetonate in acetonitrile (0.04 mol/L solution, see Fig. 1b). The deposition was carried out at 550 °C and samples were left on the hot plate until the substrate temperature reached room temperature. A solution of MAPbI_3_-DMF (1.8 mol/L) in DMF was spin-coated at 1000 rpm for 10 s followed by 5000 rpm for 30 s. During the spin coating, toluene dripping was conducted inside a N_2_ filled glove box. The film was annealed at 60 °C for 10 min and another 100 °C for 10 min. An inorganic electron acceptor layer was prepared by spin-coated at 1000 rpm for 60 s from 50 μL, ZnO colloidal solution (at room temperature) on top of the perovskite films. Then, the solution of BCP in CH_3_OH (1.0 mg/mL) was filtered through using a 0.45 µm cellulose acetate membrane filter, subsequently, was spin-coated at 1000 rpm for 60 s. Finally, Ag back contact layer was coated by thermal evaporation.

#### Measurements

Dynamic light scattering (DLS) measurements were performed using a Malvern Instruments Zetasizer Nano ZS instrument equipped with a He–Ne laser (4 mW at 633 nm). Measurements were taken at a scattering angle of 173°. XRD patterns were recorded on a Rigaku MiniFlex2 diffractometer working with Cu Kα radiation.

The photocurrent–voltage (*J*–*V*) characteristics of the perovskite solar cells were measured on a B2901A (Agilent Technologies Inc.) source meter under irradiation of AM 1.5, 100 mW/cm^2^ (1 sun) supplied by a solar simulator (YSS-80, Yamashita Denso Co., Ltd.). The incident light intensity was calibrated with a reference Si solar cell (BS-500BK, Bunkoukeiki Co., Ltd.). The active areas of the solar cells were determined with a 0.3 cm × 0.3 cm black mask. ZnO powders for x-ray diffraction (XRD) characterization were dried on the hot plate at 80 °C. For DLS measurements to measure the size of ZnO nanoparticles, the concentration was diluted to 25 times using isopropanol.

## Results and discussion

**Fig. 2:**
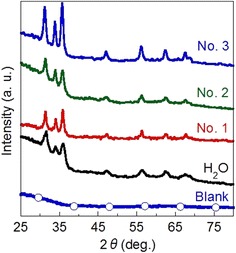
XRD patterns of ZnO nanoparticles

In order to confirm the crystal structure of the synthesized nanoparticles, the compound powder pattern was confirmed using XRD and are shown in Fig. 2. The characteristic peaks obtained for ZnO nanoparticles exist at 31.3, 33.9, 35.8, 47.1, 56.2, 62.4 and 67.6 correspond to the crystal planes of (100) (002) (101) (102) (110), (013) and (112), respectively. This result clearly indicates that ZnO nanoparticles were obtained to the ZnO hexagonal wurtzite structure [29–32]. From the results of XRD, it was confirmed that synthesis of ZnO nanoparticles was successful using both synthetic routes. However, due to the generated large amount of noise, calculation of the size of ZnO nanoparticles using XRD could not be performed.

**Fig. 3:**
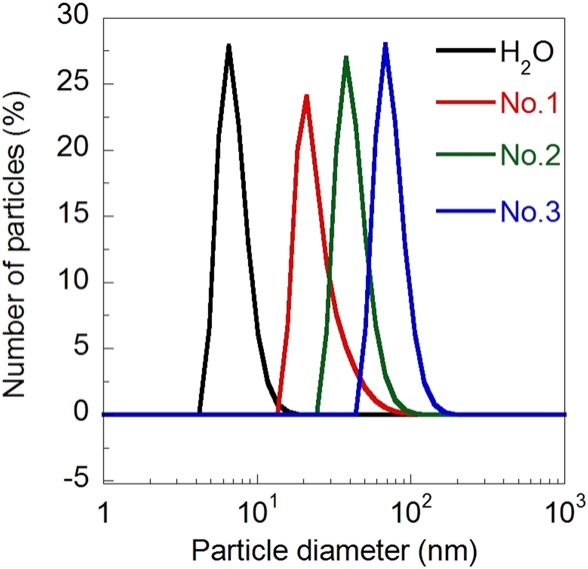
Number-distributions of hydrodynamic diameter for ZnO nanoparticles estimated with dynamic light scattering measurements

In order to confirm the particle diameter of each synthesized ZnO nanoparticles, a DLS was used and is shown in Fig. 3. Following the results of DLS, the particle diameters of each different process synthesized ZnO nanoparticles were calculated. The calculated diameter for aqueous process H_2_O, Non-aqueous process No. 1, No. 2 and No. 3, exist for 7, 25, 42 and 75 nm, respectively.

**Fig. 4:**
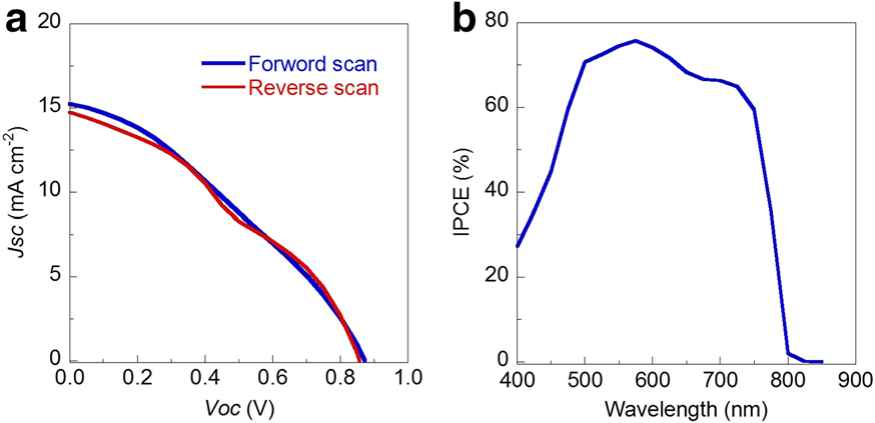
**a** *I*–*V* curves and **b** IPCE spectra measured of fabricated all inorganic perovskite solar cell using the ZnO nanoparticles ETL having diameter of 25 nm

Photovoltaic parameters and conversion efficiency of the all inorganic inverted perovskite solar cells using synthesized four ZnO nanoparticles are summarized in Table 1 and Fig. 4. In the case of incorporating ZnO nanoparticles ETL prepared by a non_aqueous process, the performance of the perovskite solar cell was relatively high for the small diameter ZnO nanoparticle. As a result, fabricated all inorganic perovskite solar cell using the ZnO nanoparticles ETL having the diameter of 25 nm, the conversion efficiency was obtained 4.3%, current–voltage (*I*–*V*) curves and incident photon to current conversion efficiency (IPCE) spectra of this cell are shown in Fig. 4. It was found that with the decrease of ZnO nanoparticles size the short-circuit current density (*Jsc*) value greatly increases and open circuit photovoltage (*Voc*) value slightly increases. When the ZnO nanoparticles with a large diameter (75 nm) were used as ETL, the *Jsc* value and *Voc* value show degradation and low conversion efficiency of 0.228% were observed. From the above results, the *Jsc* value can be increased with the decrease of the diameter ZnO nanoparticle. The reason for this may be that the use of smaller particles increases the surface area and improves the charge collection capability [33].

**Table 1:** Performance of perovskite solar cell using synthesized ZnO nanoparticles

Sample	Synthesis method	Scan direction	*Jsc* (mA/cm^2^)	*Voc* (V)	*FF* (–)	*η* (%)	Particle diameter (nm)
H_2_O	Aqueous process	Forward	7.95	0.905	0.233	1.68	7
		Reverse	10.1	0.867	0.136	1.19	
1	Non-aqueous process	Forward	15.2	0.873	0.330	4.39	25
		Reverse	14.7	0.850	0.336	4.25	
2		Forward	11.1	0.841	0.357	3.33	42
		Reverse	10.2	0.845	0.376	3.25	
3		Forward	0.871	0.405	0.453	0.160	75
		Reverse	1.10	0.417	0.645	0.296	

Scan direction: forward *J*_*SC*_ → *V*_*OC*_ and reverse: *V*_*OC*_ → *J*_*SC*_. Cell active area is 3 × 3 mm^2^ defined by a black mask

**Fig. 5:**
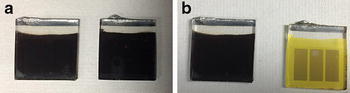
Photographs of the perovskite solar cells; **a** the fresh and **b** under the N_2_ glove box for 24 h. On the *left side*; the solar cells were fabricated using non-aqueous process synthesis method prepared ZnO nanoparticles (No. 1). On the *right side*; the solar cells were fabricated using aqueous process synthesis method prepared ZnO nanoparticles

Finally, in order to confirm the influence of synthesis route ZnO nanoparticles, the solar cells were fabricated using aqueous process synthesis method prepared ZnO nanoparticles as ETL. The perovskite solar cells were left for a day under the N_2_ glove box and the photograph of the fresh and aged cells are shown in Fig. 5. Despite the N_2_ atmosphere with very little moisture, the perovskite solar cells using ZnO nanoparticles ETL prepared by aqueous synthesis process changed its colour from black to yellow in a day. In our previous study, it is clarified that this observed change in the colour is resulting from the decomposition of the perovskite layer by moisture/water [34]. This may result from the synthesis process evolved residue water in fabricated ZnO nanoparticles. The low *Jsc* value is thought to be due to the effect of residue water emanating from the aqueous synthesis process.

## Conclusions

In conclusion, we fabricated all inorganic inverted perovskite solar cells using synthesized ZnO nanoparticles and confirmed the optimum size of the ZnO nanoparticles and the superiority of the synthetic route. We found that *Jsc* and *Voc* value improves by decreasing the size of ZnO nanoparticles. Although *Voc* value is not affected by the influence of residual water in the solution for preparing the ZnO layer, *Jsc* value is decreased. As a result, we found two guidelines for manufacturing inorganic perovskite type solar cells with high efficiency; (1) use of oxide nanoparticles with small particle size; (2) reduction of moisture content in oxide forming material. In this experiment, it was impossible to understand the factor given to the fill factor (*FF*) value. For that reason, we are currently searching for factors that influence the value of *FF* value by performing internal interface analysis [35–37].
